# *Fasciola hepatica* Surface Tegument: Glycoproteins at the Interface of Parasite and Host*[Fn FN2]

**DOI:** 10.1074/mcp.M116.059774

**Published:** 2016-07-27

**Authors:** Alessandra Ravidà, Krystyna Cwiklinski, Allison M. Aldridge, Paul Clarke, Roisin Thompson, Jared Q. Gerlach, Michelle Kilcoyne, Cornelis H. Hokke, John P. Dalton, Sandra M. O'Neill

**Affiliations:** From the ‡Fundamental and Translational Immunology, School of Biotechnology, Faculty of Science and Health, Dublin City University, Glasnevin, Dublin 9, Ireland;; §School of Biological Sciences, Medical Biology Centre (MBC), Queen's University Belfast, Belfast, Northern Ireland, UK;; ¶Glycoselect, Dublin City University, Glasnevin, Dublin 9;; ‖Glycoscience Group, National Centre for Biomedical Engineering Science, National University of Ireland Galway, Ireland;; **Regenerative Medicine Institute, NUI Galway, Ireland;; ‡‡Carbohydrate Signalling Group, Microbiology, NUI Galway, Ireland;; §§Department of Parasitology, Leiden University Medical Center, Leiden, The Netherlands

## Abstract

*Fasciola hepatica*, commonly known as liver fluke, is a trematode that causes Fasciolosis in ruminants and humans. The outer tegumental coat of *F. hepatica* (FhTeg) is a complex metabolically active biological matrix that is continually exposed to the host immune system and therefore makes a good vaccine target. *F. hepatica* tegumental coat is highly glycosylated and helminth-derived immunogenic oligosaccharide motifs and glycoproteins are currently being investigated as novel vaccine candidates. This report presents the first systematic characterization of FhTeg glycosylation using lectin microarrays to characterize carbohydrates motifs present, and lectin histochemistry to localize these on the *F. hepatica* tegument. We discovered that FhTeg glycoproteins are predominantly oligomannose oligosaccharides that are expressed on the spines, suckers and tegumental coat of *F. hepatica* and lectin blot analysis confirmed the abundance of N- glycosylated proteins. Although some oligosaccharides are widely distributed on the fluke surface other subsets are restricted to distinct anatomical regions. We selectively enriched for FhTeg mannosylated glycoprotein subsets using lectin affinity chromatography and identified 369 proteins by mass spectrometric analysis. Among these proteins are a number of potential vaccine candidates with known immune modulatory properties including proteases, protease inhibitors, paramyosin, Venom Allergen-like II, Enolase and two proteins, nardilysin and TRIL, that have not been previously associated with *F. hepatica*. Furthermore, we provide a comprehensive insight regarding the putative glycosylation of FhTeg components that could highlight the importance of further studies examining glycoconjugates in host-parasite interactions in the context of *F. hepatica* infection and the development of an effective vaccine.

Fasciola hepatica is a parasitic flatworm of livestock and the causative agent of Fasciolosis, a disease that results in major economic losses to the agricultural industry globally, estimated at $3 billion annually (1). Fasciolosis has recently been acknowledged by the World Health Organization (WHO) as a neglected tropical zoonotic disease, with as many as 2 to 17 million people infected worldwide and 180 million at risk of infection (2). Infection in animals is currently treated chemically with drugs such as triclabendazole, albendazole, and oxyclosanide, although drug-resistance is now widespread across Europe and globally (3). Although vaccines are considered a safe, economically viable and environmentally friendly solution none have been commercially developed to date and there is a dearth of promising candidates with potent immunoprotective efficacy in the pipeline (4).

It is only recently that the fluke surface tegument has been the subject of close investigations by molecular techniques (4, 5, 6, 7) with the aim of discovering novel potential targets for both drug and vaccine development. The *F. hepatica* tegument is a metabolically active layer that is continuously sloughed off and replaced during infection. The tegument is intimately associated with host tissues and performs a number of important functions including the synthesis and secretion of substances, absorption of nutrients, osmoregulation, and protection against host enzymes (8). Closely packed spines that point posteriorly are distributed throughout the tegument and help maintain the position of the fluke within the tissues and bile duct. The spines also facilitate feeding of the obligate blood-feeding adult through the erosion of the epithelium and puncturing of small bloods vessels (8). It is highly likely that the shed tegumental coat is released into the blood stream and studies have shown the presence of anti-tegumental antibodies in serum form infected animals (9).

The outer-most surface of the tegument is shielded by a glycocalyx that is comprised predominantly of glycoconjugates (10). Glycoproteins and glycolipids of parasitic helminths often contain a mixture of oligosaccharide motifs similar or identical to those present in their hosts, as well as structurally-unusual, pathogen-specific motifs (11). These glycoconjugates can play key roles in the immunoregulatory activity of the parasite by interacting with C-type lectin receptors (CLRs)^1^ (12). We have previously shown that a preparation of tegumental antigen (FhTeg) exhibits potent Th1 immune suppressive properties *in vivo* by reducing serum levels of IFNγ and IL-12p70 in the mouse model of septic shock (13). FhTeg-activated dendritic cells and mast cells are hypo-responsive to Toll-like Receptors (TLR) activation and thereby suppress the production of inflammatory cytokines and co-stimulatory molecules important in driving adaptive immune responses (14). The FhTeg mechanism of action is independent of TLRs and has been linked to the suppression of NF-κB and MAPK pathways and enhanced expression levels of suppressor of cytokine signaling (SOCS) 3, a negative regulator of the TLR and STAT3 pathways that is observed *in vitro* and following *F. hepatica* infection (15, 16).

The contribution of glycoconjugates to the development of protective immunity to *Fasciola* infection has yet to be established and, even if these oligosaccharides are not antigenic *per se*, they can influence the immunogenicity of glycoprotein-based vaccines by contributing to correct protein folding and biophysical properties (17), as well as to the modulation of antigen processing and activation of antigen presenting cells. One of the first steps of understanding the role of *Fasciola* glycoconjugates is to identify the glycoproteins on its tegumental coat. Here we employed orthogonal lectin assays to probe the *F. hepatica* tegumental components and profile the variety of glycoprotein and carbohydrate moieties at or close to the parasite surface. Selective enrichment of these tegumental glycoproteins was performed using lectin chromatography and these were subjected to proteomic analysis. This study provides a comprehensive and novel insight regarding glycoprotein composition of the FhTeg and has identified those that likely play critical functions at the interface between parasite and host.

## MATERIALS AND METHODS

### 

#### 

##### Antigen Preparation

*F. hepatica* tegumental extract (FhTeg) was prepared as previously reported (13). In brief, *F. hepatica* adult worms following collection from sheep at an abattoir in Ireland were washed in sterile phosphate-buffered saline (PBS) and incubated in 1% Nonidet P-40 (Nonidet P-40 (Sigma Aldrich, Wicklow, Ireland) in PBS) for 30 min. Supernatant was collected and Nonidet P-40 removed using detergent-removing biobeads (Bio-Rad laboratories, Fannin Ltd, Dublin, Ireland), and the remaining supernatant was centrifuged at 14,000 × *g* for 30 min at 4 °C prior to being filtered/concentrated using compressed air, and then stored at −20 °C. All protein concentrations were determined using a bicinchoninic acid protein assay kit (Promeaga, Madison, WI).

##### Profiling of FhTeg Oligosaccharides by Lectin Microarray

FhTeg was fluorescently labeled with Alexa Fluor 555 *via* carboxylic acid succinimidyl ester conjugation strategy according to manufacturer instructions (ThermoFischer Scientific. MSc Co ltd, Dublin, Ireland). Excess dye was removed from FhTeg-555 using a desalting column (7 kDa molecular weight cut off (MWCO) (Invitrogen, Paisley, UK)). Final protein concentration and labeling efficiency was calculated according to the manufacturer's instructions by measuring sample absorbance at 280 and 550 nm and using the arbitrary values for molecular mass (100,000) and extinction coefficient (E of 10).

A panel of 48 lectins (supplemental Table S1) was printed on Nexterion® Slide H microarray slides in a 62% (±2%) humidity environment using a SciFLEXARRAYER S3 (Scienion AG, Berlin, Germany) equipped with a 90 μm uncoated glass dispenser capillary to construct the lectin microarray as previously described (18). Microarray incubations were carried out essentially as previously described (18) and all procedures were carried out in the dark. In brief, FhTeg-555 was diluted in Tris-buffered saline supplemented with Ca^2+^ and Mg^2+^ ions (TBS; 20 mm Tris-HCl, 100 mm NaCl, 1 mm CaCl_2,_ 1 mm MgCl_2_, pH 7.2) and with 0.05% Tween 20 (TBS-T) for incubation on the microarrays using an eight-well gasket (Agilent Technologies, Cork, Ireland) at room temperature for 1 h with gentle rotation (4 rpm) in the dark. Initially several concentrations (2.9–14.4 μg/ml) were titrated to determine the best concentration for optimal signal to noise ratio. Based on the binding interactions observed from the titration experiments, samples were also coincubated with 100 mm Gal and Man in parallel on the lectin microarrays to verify carbohydrate-mediated binding (19). Incubations were carried out in triplicate using the optimal concentration of 8.6 μg/ml FhTeg in TBS-T. After incubation, the microarray slides were disassembled under TBS-T, washed three times in TBS-T for 2 min each with gentle agitation in a Coplin jar, with a final 2 min wash in TBS. The slides were centrifuged until dry and scanned immediately with the 532 nm laser (90% laser power, 5 μm resolution) of an Agilent G2505B microarray scanner. Three replicate experiments were performed.

Microarray data extraction was performed as previously described (18). In brief, raw intensity values were extracted from the image files using GenePix Pro v6.1.0.4 (Molecular Devices, Berkshire, UK) and a proprietary address file (*.gal) using adaptive diameter (70–130%) circular alignment based on 230 μm features and exported as text to Excel (version 2007, Microsoft). Local background-corrected median feature intensity data (F543median-B543) was selected and the median of six replicate spots per subarray was handled as a single data point for graphical and statistical analysis. Data were normalized to the mean of three replicate microarray slides (subarray by subarray using subarray total intensity), and binding data was presented in histogram form of mean intensity with one standard deviation of three experimental replicates (*n* = 3, 18 data points in total). Unsupervised clustering of normalized lectin binding data was performed with Hierarchical Clustering Explorer v3.5 (HCE 3.5; Human-Computer Interaction Lab, University of Maryland, http://www.cs.umd.edu/hcil/hce/hce3.html). Mean normalized data was initially clustered with the following parameters: no prefiltering, complete linkage and Euclidean distance.

##### SDS-PAGE, Lectin and Western Blot Analysis of FhTeg

Precast 10% sodium dodecyl sulfate (SDS)-polyacrylamide gels (Thermo Fisher Scientific) were run under standard reducing conditions. Samples were loaded at a concentration of 10 μg/ml and gels were subjected to Coomassie or silver staining (20), or transferred onto nitrocellulose membranes by iBlot® Dry blotting system (Thermo Fisher Scientific) for Western analysis. After standard Western blotting procedures with primary (goat anti-biotin antibody and biotinylated lectins as per manufacturer's instructions (Vector Laboratories Ltd, Peterborough, UK) and secondary antibody (IRDye 800 Streptavidin and IRDye 680 anti-goat; Li-COR Biosciences, Lincoln, NE), the membranes were scanned using Odyssey Infrared Imaging System (Li-COR Biosciences). Data analysis was performed with Odyssey V 3.0 software (Li-COR Biosciences).

##### Lectin- and Immunofluorescence Microscopy of Parasite Surface Oligosaccharides

Adult liver flukes were flat-fixed in 4% paraformaldehyde and incubated with fluorescein-labeled lectins or with biotin-MAL II followed by incubation with fluorescein-labeled anti-biotin (Vector Laboratories). Parasites were mounted on glass microscope slides with Vectashield® anti-fading solution with or without DAPI (Vector Laboratories). Specimens were viewed using a Leica DM IL LED microscope using 10×, 20×, and 40× HI PLAN I objectives (Leica Microsystems, Ashbourne, Ireland) equipped with epifluorescent source and a filter system for FITC and TRITC fluorescence. Images were processed with Adobe Photoshop CS4 software (Adobe System Inc. San Jose, CA).

##### Selective Biotinylation and Isolation of FhTeg Glycoproteins

FhTeg glycoproteins (Glyco-FhTeg) were isolated by biotinylating the carbohydrates with carbohydrate-specific EZ-link hydrazide-biotin (Thermo Fisher Scientific) according to manufacturer guidelines (see supplemental Fig. S1*A*). Western blot analysis with IR-labeled streptavidin complex confirmed the efficient biotinylation of the FhTeg glycoproteins (FhTeg-B; supplemental Fig. S1*B*). FhTeg-B was purified using the biotin/avidin system using a monomeric avidin-agarose column (Thermo Scientific). Nonbiotinylated (nonglycosylated) proteins flowed through the column constituting the Avidin FT fraction, whereas biotinylated (glycosylated) proteins (Avidin B) were displaced from avidin-immobilized beads by elution with concentrated biotin in reducing conditions. This purified biotinylated glycoprotein fraction is referred to as Glyco-FhTeg. Western blot analysis (supplemental Fig. S1*C*) confirmed the presence of biotinylated molecules in the starting material (FhTeg-B), bound fraction (FhTeg-B Avidin B) but not in the flow through material (FhTeg-B Avidin FT).

##### Isolation of Mannose-rich Glycoproteins by Lectin Affinity Chromatography

Mannose-rich glycoprotein components of the FhTeg were obtained by lectin affinity chromatography using plant lectins ConA, LCA, and GNL. FhTeg was buffer exchanged in lectin binding buffer (20 mm Tris-HCl, 0.15 m NaCl, 1 mm CaCl_2_, 1 mm MnCl_2_, pH 7.2) using 7 kDa MWCO Zeba spin columns (Thermo Fisher Scientific) and then incubated with ConA-, LCA-, or GNL-agarose slurry (Vector Laboratories ltd) with end-over-end agitation overnight at 4 °C. The beads were centrifuged and the unbound fractions decanted. Following extensive washing, the beads were incubated for 3 h at room temperature with two column volumes of inhibiting sugars to elute bound oligosaccharides (0.5 m *alpha*-methylmannoside and 0.5 m alpha-methylglucoside for ConA; 0.5 m methylmannoside for GNL and LCA). High concentrations of salts and monosaccharides were removed from both unbound and bound fractions by diafiltration in Amicon Ultra centrifugal filters with 3 kDa MWCO (Millipore Ireland B.V., Cork, Ireland). Isolation yields and efficiency were monitored by measuring total protein content of fractions with BCA assay, SDS-PAGE and lectin blot probed with ConA, LCA, and GNL (supplemental Fig. S1), as described above, before subjecting these to proteomic analysis. The combined proteome of the three lectin affinity purified fractions (ConA, LCA, and GNL) is referred to Man-FhTeg.

##### Experimental Design and Statistical Rationale

Microarray, lectin blots, and micrographs were performed with at least two biological replicates. For the proteomics study, whole *Fasciola* tegument was initially analyzed by LC-MS/MS. Because the aim of this experiment was to profile the molecules associated with fluke tegument, no biological replicates were used and therefore no quantitation was performed.

##### Mass Spectrometry Analysis of FhTeg lectin-purified Fractions

Selected *F. hepatica* fractions were subjected to proteomics analysis by mass spectrometry (MS by Proteomique Platform of the Quebec Genomics Center, CHU de Quebec Research Centre, Quebec, Canada). Liquid chromatography/tandem mass spectrometry (LC-MS/MS) was carried out according to a previously reported protocol (21). Briefly, proteins were resolved by SDS-PAGE, the gel was stained with SYPRO Ruby gel stain according to the manufacturer's instructions (Bio-Rad Laboratories). The protein bands were cut into gel slices and deposited in 96-well plates. A liquid handling station (MassPrep; Waters, Dublin, Ireland) was used, with sequencing-grade modified trypsin (Promega, Madison, WI) for in-gel protein digestion according to the manufacturer's instructions. Peptide extracts were then dried by evaporation in a SpeedVac (Thermo Fisher Scientific). LC-MS/MS experiments were performed with an TripleTOF 5600 mass spectrometer connected to a Thermo Surveyor MS pump and equipped with a nanoelectrospray ion source (all from Thermo Fisher Scientific). The peptides were separated in a PicoFrit BioBasic C18 column (0.075 mm I.D. x 10 cm; New Objective), with a linear gradient from 2 to 50% solvent B (acetonitrile, 0.1% formic acid) over 30 min at a flow rate of 200 nl/min. Mass spectra were acquired in the data-dependent acquisition mode (Xcalibur software, version 2.0). Each full-scan mass spectrum (*m*/*z* 400–2000) was followed by the collision-induced dissociation of the seven ions giving the most intense signals. The dynamic exclusion function was enabled (30 s of exclusion), and the relative collisional fragmentation energy was set at 35%. Peak list files were generated by Protein Pilot v5.0 (SCIEX, co AB Sciex UK Limited, Cheshire, UK) using default parameters and exported to Mascot v2.4.1 (Matrix Science, Zürich, Switzerland) for database searching.

##### Database Searching

All MS/MS spectra were analyzed with Mascot (version 2.4.1; Matrix Science), set up to search against a database comprised of gene models identified from the recently sequenced *F. hepatica* draft genome (version 1.0, 101,780 entries; 22) and the Uniprot sheep protein data bank (27,174 entries), assuming digestion with trypsin with two missed cleavages permitted. A subset of this database was used for the tandem spectra also assuming trypsin. The *F. hepatica* gene model sequences can be accessed through WormBase ParaSite (http://parasite.wormbase.org/) under accession PRJEB6687 (genomic read data and gene model transcripts). Fragment and parent ion mass tolerance were set at 0.100 Da. Carbamidomethylation of cysteine was as a fixed modification and the dehydration of the N terminus, Glu->pyro-Glu of the N terminus, ammonia-loss of the N terminus, Gln->pyro-Glu of the N terminus, deamidation of asparagine and glutamine, oxidation of methionine, hex of arginine and threonine and biotin of lysine were specified as variable modifications.

##### Criteria for Protein Identification

Scaffold (version 4.3.4, Proteome Software Inc., Portland, OR) was used to validate MS/MS based peptide and protein identifications. Peptide identifications were accepted if they could be established at greater than 95% probability to achieve an FDR less than 1.0% by the Peptide Prophet algorithm (24) with Scaffold delta-mass correction. Protein identifications were accepted if they could be established at greater than 95.0% probability to achieve an FDR less than 1.0% and contained at least 2 identified peptides. Protein probabilities were assigned by the Protein Prophet algorithm (24). Proteins that contained similar peptides and could not be differentiated based on MS/MS analysis alone were grouped to satisfy the principles of parsimony. Putative annotation of the *F. hepatica* gene models was assigned using *in silico* tools, Uniprot, Gene Ontology (GO), and InterproScan (20). The identified proteins in this study were categorised according to their GO classification. Glycosylation predictive analysis was carried out using NetNGlyc 1.0 and NetOGlyc 4.0 Servers (25).

## RESULTS

### 

#### 

##### Lectin Microarray Profiling of the F. hepatica Tegument Demonstrates Characteristic Glycoprotein Content

**Fig. 1.**
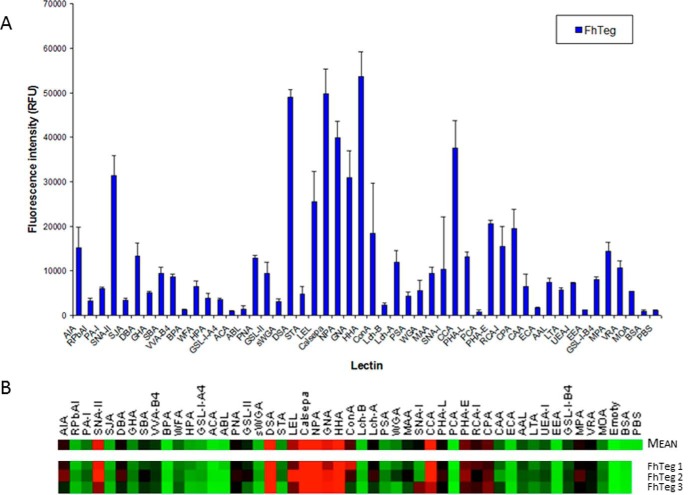
**Lectin microarray reveals that *Fasciola hepatica* tegument (FhTeg) exhibits a rich and complex oligosaccharide make up.** Fluorescently labeled FhTeg (8.6 μg ml^−1^) was incubated with a proprietary lectin microarray (1 h, 23 °C, 4 rpm) in TBS-T supplemented with 1 mm Ca^2+^ and Mg^2+^. Slides were scanned in a microarray scanner using a 543 nm laser. Histogram represents the the mean fluorescence intensity of three experimental replicates (18 data points in total) of fluorescently labeled FhTeg binding to printed lectins. Error bars are the standard deviation of the mean of three experimental replicates (*A*). Binding data is also represented as a heat map and depicts both mean intensity and individual replicates (*B*) in which little to no binding intensity is represented by green and greatest intensity by red.

The glycosylation of FhTeg was characterized on a lectin microarray featuring a comprehensive panel of plant, bacterial and fungal lectins (Fig. 1*A* and supplemental Table S1). FhTeg exhibited a particularly high affinity for the Man-binding lectins (Calsepa, NPA, GNA, HHA and Con A; supplemental Table S1), which suggested a predominance of glycoproteins modified with oligomannose type *N*-linked oligosaccharide structures at the parasite surface. Binding to LEL and DSA indicated the presence of *N*-acetylglucosamine (GlcNAc) residues, but these residues were likely predominantly part of the chitobiose core (GlcNAc-β-(1,4)-GlcNAc) of *N*-linked structures and/or *N*-acetyllactosamine (Gal-β-(1,4)-GlcNAc) in the antennae rather than terminal GlcNAc residues as other typical GlcNAc-binding lectins such as GSL-II displayed only moderate binding. FhTeg also bound to the fucose-binding lectins AAL, LTA and UEA-I and to the terminal α-linked galactose-(Gal-) binding lectins GSL-I-B4, MPA, VRA, and MOA. The presence of complex-type *N*-linked oligosaccharides was also confirmed by binding to PHA-L and -E and CPA (Fig. 1A). Structures with terminal β-linked Gal and residues were inferred to be relatively abundant because of moderate binding intensity with the lectins RCA-I, AIA, PNA, and PHA-E with *N*-acetylgalactosamine (GalNAc) residues further indicated by more intense binding with SNA-II (supplemental Table S1). Additionally, the binding of AIA and PNA may further indicate the presence of O-linked oligosaccharides in FhTeg protein extract, more specifically the T antigen (Gal-β-(1,3)-GalNAc-O-Ser/Thr), and PNA binding suggested that these structures were not sialylated (supplemental Table S1). Interestingly, only low binding was observed for lectins with binding affinities for sialic acid-containing moieties, SNA-I, MAA, and WGA, except for CCA, which recognizes 9-O-acetyl- and 4-O-acetylneuraminic acids (26) and bound intensely with FhTeg (Fig. 1*A*). Only minor differences were observed between three separate analyses of the FhTeg (Fig. 1*B*).

##### Oligosaccharide Motifs are Localized Mainly at the Spines and Suckers of Adult Flukes and are Present on a Wide Panel of Glycoproteins

To determine the localization of lectin-binding motifs on the superficial aspects of the tegumental coat, flat fixed adult flukes were incubated with a panel of fluorescein-conjugated lectins selected on the basis of the above microarray data. Although all lectins yielded remarkable visualization of the external morphological features of the adult fluke such as oral and ventral suckers, tegumental spines, and tegumental coat, differences were observed in the binding patterns between lectins. Table II summarizes the micrograph observations based on the anatomical region visualized and the intensity of fluorescence.

**Table I:** Lectin origin and nominal oligosaccharide binding specificities according to CFG oligosaccharide array public data. Man: Mannose; LacNAc: N-acetyllactosamine; GlcNAc: N-acetylglucosamine; Fuc: Fucose; Gal: Galactose; GalNAc: N-acetylgalactosamine; Neu5Ac: N-acetylneuraminic acid; Thr: Threonine; Ser: Serine

Abbreviation	Origin	Binding specificity
ConA	*Concanavalia ensiformis*	α-linked Man, branched and terminal
LCA	*Lens Culinaris*	Core fucosylated terminal LacNAc, Man, GlcNAc
PSA	*Pisum sativum*	Core fucosylated terminal LacNAc, Man, GlcNAc
GNL	*Galanthus nivalis*	Man
AAL	*Aleuria aurantia*	Fuc-α-(1,6)-GlcNAc; Fuc-α-(1,3)-Gal-β-(1,4)-GlcNAc
UEA-I	*Ulex europaeus*	Fuc-α-(1–2)-LacNAc
PHA-E	*Phaseolus vulgaris*	Biantennary complex *N*-linked glycans
PHA-L	*Phaseolus vulgaris*	Tri- and tetra-antennary complex oligosaccharides
sWGA	*Triticum vulgaris*	GlcNAc
(succinylated WGA)		
Jacalin	*Artocarpus integrifolia*	GlcNAc-β-(1,3)-Gal; Gal-β-(1,3)-GalNAc-α-Thr/Ser
WGA	*Triticum vulgaris*	GlcNAc; Neu5Ac
GSL-II	*Griffonia simplicifolia*	GlcNAc
SBA	Soybean	Terminal GalNAc
DBA	*Dolichos biflorus*	α-GalNAc
VVL	*Vicia villosa*	Terminal GalNAc
GSL-I	*Griffonia simplicifolia*	α-GalNAc; GalNAc α-Thr/Ser; α-Gal
MAL-II	*Maackia amurensis*	Neu5Ac-α(2,3)-Gal(NAc); sulfated glycans
MAL-I	*Maackia amurensis*	LacNAc; Neu5Ac-α-(2,3)-Gal(NAc)
SNA-I	*Sambucus nigra*	Neu5Ac-α-(2,6)-Gal(NAc)
PNA	*Arachis hypogea*	Gal-β-(1,3)-GalNAc-α-Thr/Ser
RCA_120_	*Ricinus communis*	LacNAc
ECL	*Erythrina cristagalli*	LacNAc

**Table II:** Summary of lectin binding on micrographs. + to ++ low intensity; +++ medium intensity; +++++ high intensity

Lectin	Spines/Spinlets	Suckers Oral/Ventral	Surface coat
Mannose binding lectins			
ConA	+++++	+++++	+++++
LCA	+++++	+++++	+++++
PSA	+++++	+++++	+++++
GNL	+++++	+++++	+++++
Fucose binding Lectin			
AAL	+++++		
UEA	+		+++++
Complex-type N-Linked oligosaccharides binding lectins			
PHA-E		+++++	
PHA-L		+++++	
Terminal GlcNAc binding lectins			
S-WGA	+++++	+++++	+++++
Jaclin	+++++	+++++	+++++
WGA	+++++	+++++	+++++
GSL-I	+++++	+++++	+++++
Terminal GalNAC binding lectins			
SBA	+++++	+++++	+++++
DBA	++	++	++
VVL	++	++	++
GSL-1	+++++	+++++	+++++
Terminal α2–3 sialic acids binding lectins			
MAL-II	+++		
MAL-I	+++		
SNA	+++		
Terminal Galactose binding lectins			
PNA	+++++		+++
RCA	_____		+++
ECL	+++++		+++

**Fig. 2.**
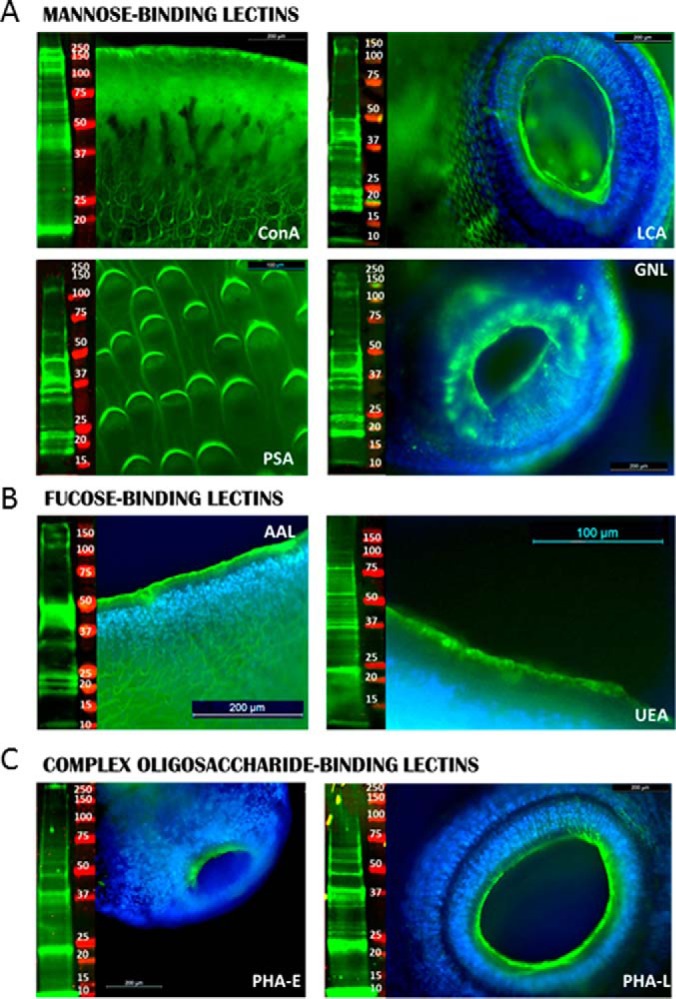
**Mannose- and fucose-rich glycoproteins are highly abundant and predominantly localized on scales and suckers of adult flukes.** Flat fixed whole adult flukes were probed with fluorescein-labeled mannose, fucose and complex oligosaccharide-binding lectins (green) and, where displayed, with DAPI nuclear staining (blue). Lectin blots represent tegumental proteins (15 μg), probed with mannose, fucose and complex oligosaccharide biotin-labeled lectins orIR-labeled streptavidin complex (green), molecular mass marker (red).

All Man-binding and terminal GlcNAc-binding Lectins Shared a Strong Binding to Fluke Spines, Sucker, and Tegumental Coat (Fig. 2*A*; 3*A*). Similarly, all GalNAc-binding lectins bound to these anatomical features, with strong binding being observed for SBA and GSL-I compared with weaker binding for VVL and DBA. AAL, which recognizes predominantly α-(1,3) and α(1,6)-linked Fuc core modifications, predominantly bound to the tegumental spines, compared with UEA-I, which was predominantly observed at the surface edges of the fluke's coat, suggesting a widespread but low expression of the α(1–2)-linked Fuc moieties recognized by UEA-I. The complex-type *N*-linked oligosaccharides that were selectively bound by PHA-E and PHA-L were only observed at the edge of the sucker areas. Binding with affinity for α(2–3)-linked sialic acids (MAA) and Gal (PNA and ECL) were primarily observed on the spines and spinelets.

**Fig. 3.**
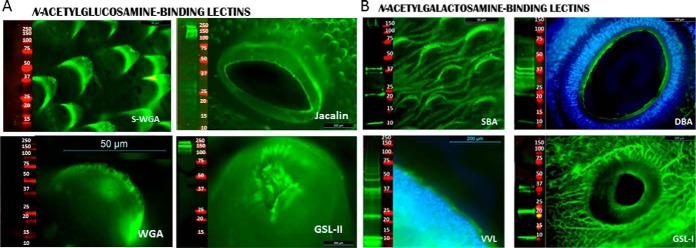
**GlcNAc-rich motifs are efficiently recognized by lectin fluorescence on adult flukes but not by lectin blots.** GlcNAc- and GalNAc-rich glycoconjugates are highly expressed on FhTeg as visualized in lectin fluorescence micrographs but only a few protein bands were recognized by lectin blots of adult *Fasciola hepatica* tegumental coat. Flat fixed whole adult flukes were probed with fluorescein-labeled GlcNAc- and GalNAc-binding lectins (green) and, where displayed, with DAPI nuclear staining (blue). In lectin blots, tegumental proteins (15 μg) were probed with GlcNAc- and GalNAc-binding biotin-labeled lectins or IR-labeled streptavidin complex (green), molecular mass marker (red).

**Fig. 4.**
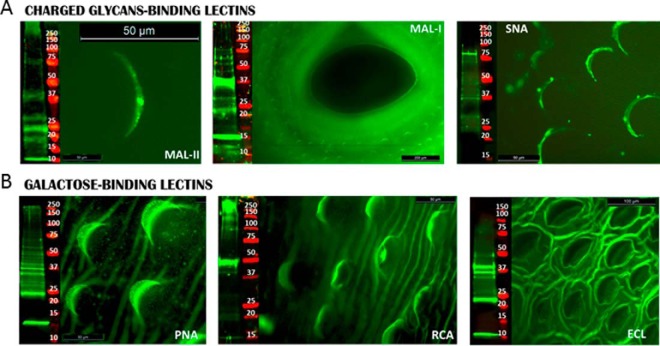
**Negatively charged oligosaccharides are present on scales and suckers of adult flukes.** Flat fixed whole adult flukes were probed with fluorescein-labeled charged and galactose binding lectins (green) and, where displayed, with DAPI nuclear staining (blue). In lectin blots, tegumental proteins (15 μg) were probed with charged and galactose biotin-labeled lectins/IR-labeled streptavidin complex (green), molecular mass marker (Red).

The same panel of lectins was also used to probe blots of denatured FhTeg protein extracts (Figs. 2–4). ConA bound to multiple glycoproteins and bound to the greatest number of glycoproteins of all the lectins examined and the greatest *M*_r_ range (Fig. 2*A*). LCA and PSA that bind to oligomannose oligosaccharides and GNL that recognizes structures containing α(1–3)linked Man residues, bound to proteins of similar *M*_r_ but to a lower number of proteins. Differences were also observed for the Fuc-binding lectins; AAL bound intensely to a small number of glycoprotein bands at ∼20–25, 32, 37–47 kDa, compared with a wider *M*_r_ range of protein bands weakly bound by UEA-I. PHA-E and PHA-L, bound to four main protein bands at ∼10, 22, 38, and 48 kDa, with some additional minor bands between 10 to 75 kDa (Fig. 2*C*).

By contrast, only two prominent protein bands, at ∼150 and 200 kDa (Fig. 3*A*), were detected by Jacalin and GSL-II. GSL-II binds to GlcNAc residues whereas Jacalin can bind to terminal β-linked Gal (which can be present on *N*-linked structures as well as O-linked). Jacalin has the greatest affinity for T antigen, but does not conclusively prove its presence whereas Jacalin may indicate the presence of O-linked structures, in agreement with the PNA and AIA binding observed on the lectin microarray, both of which bind only O-glycosidically linked oligosaccharides (Fig. 3*A*). No proteins were detected by WGA (recognizes GlcNAc and Neu5Ac motifs) and sWGA (recognizes GlcNAc motifs), which suggest that the structures histochemically recognized by these lectins could be present on glycolipids. All GalNAc-binding lectins detected a similar *M*_r_ range by lectin blots, with dominant binding to protein bands observed at 10, 22, and 37 kDa (Fig. 3*B*). VVL and DBA bind to proteins between the *M*_r_ range of 50 to100 kDa.

MAL II-binding motifs were present on a number of glycoproteins at ∼10, 17, 22, 40, 75kDa and >250 kDa (Fig. 4*A*). Similarly MAL-I motifs were found at this *M*_r_, supporting the possible presence of α(2–3)-linked sialic acid. SNA-I that recognizes α(2–6)-linked sialic acid bound to proteins at an *M*_r_ range between 20 to 75 kDa (Fig. 4*A*). The binding to MAL I and SNA motifs suggests that both terminal and nonterminal sialylated oligosaccharides are present. The abundance of LacNAc was confirmed by the strong binding observed for all the Gal-binding lectins used in this study (PNA, RCA-I and ECL; Fig. 4*B*). Terminal β-linked Gal, including the residue in LacNAcis a common feature on the antennae of complex and hybrid *N*-linked mammalian-oligosaccharides, as well as on O-linked oligosaccharides. PNA and ECL both bound to single bands at ∼10 and 22 kDa and a number of protein bands at ∼37 kDa. Fewer protein bands were detected by RCA-I at 50 and between 150–250 kDa (Fig. 4*B*).

##### Proteomic Analysis FhTeg Glycosylated and Mannosylated Protein Subsets

**Fig. 5.**
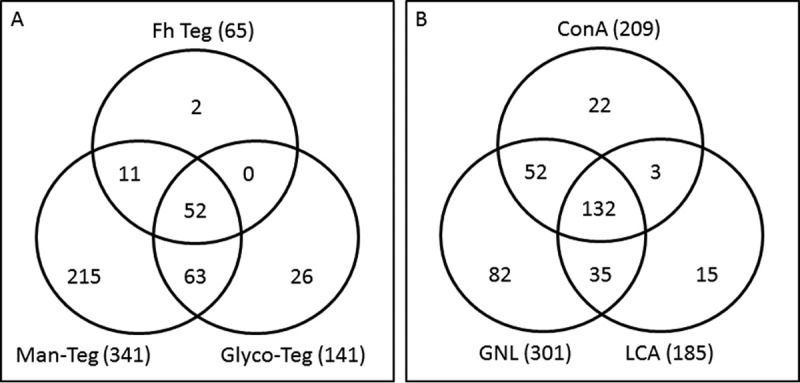
Venn diagram to demonstrate (*A*) the number of proteins identified within the *F. hepatica* tegument (FhTeg) and the lectin enriched glycoprotein fractions of the tegument (Glyco-FhTeg and Man-FhTeg) and (*B*) the number of proteins identified within the mannose lectin enriched glycoprotein fraction of the tegument (Man-FhTeg).

A total of 369 proteins were identified by proteomic analysis of FhTeg and two subfractions isolated using biotinylation of the tegument, Glyco-FhTeg, and mannose-lectin affinity chromatography, Man-FhTeg. Interestingly, despite Glyco-FhTeg and Man-FhTeg being glycoprotein subsets of FhTeg, during parallel analysis more proteins were identified in these fractions (*n* = 141 and 341, respectively) than in FhTeg (*n* = 65) (Fig. 5*A*). We believe that elimination of major nonglycosylated proteins from the FhTeg by the enrichment of specific subsets by affinity chromatography enabled the identification of broader profile of glycoproteins. Analysis of the *F. hepatica* tegumental proteins against the Uniprot sheep protein data bank, resulted in 28 protein identifications, with the majority of the proteins identified enriched within the FhManTeg sample. These proteins were associated with the host blood and immune systems, including serum albumin, hemoglobin, hemopexin, IgM, IgJ, and alpha 2 macroglobulin. The full lists of proteins identified are reported in supplemental Tables S3–S4 Not surprisingly, however, the vast majority the proteins identified in FhTeg were shared with the Glyco-FhTeg and Man-FhTeg preparations, confirming the high level of glycosylation of *F. hepatica* tegumental proteins. Dynein 8 kDa light chain outer arm and glutathione S-transferase mu class 27 were the only two protein hits unique to FhTeg. Comparative analysis of the 141 biotinylated glycoproteins (Glyco-FhTeg) with the 341 mannosylated glycoproteins (Man-FhTeg) revealed that 26 glycoproteins were unique to the Glyco-FhTeg preparation (Table III) and that these comprised of mainly glycoproteins important for protein synthesis and structure and a number of catalytic enzymes.

**Table III:** Unique proteins identified from the GlycoFhTeg preparation annotated using the F. hepatica genome, grouped by Gene Ontology classification. * UP–unique peptide

Description	Identifier	UP
*Molecular Function: Binding (GO:0005488)*		
Actin	BN1106_s3541B000067	3
Actin	BN1106_s2907B000132	2
Actin interacting protein 1	BN1106_s2434B000197	2
Annexin	BN1106_s819B000365	2
Elongation factor 2	BN1106_s1739B000159	3
Eukaryotic initiation factor 4A	BN1106_s664B000642	4
HSP70	BN1106_s617B000558	3
Titin	BN1106_s1119B000202	2
Uncharacterized protein	BN1106_s538B000488	3
*Molecular Function: Catalytic activity (GO:0003824)*		
Alanine aminotransferase	BN1106_s1834B000180	2
Aldo-keto reductase	BN1106_s1026B000549	5
Aldose 1-epimerase	BN1106_s4504B000167	2
cAMP-dependent protein kinase	BN1106_s417B000229	2
Dipeptidyl peptidase 3	BN1106_s13034B000002	2
Glucan/glycogen phosphorylase	BN1106_s916B000191	3
Inorganic pyrophosphatase	BN1106_s1848B000328	2
Retinol dehydrogenase	BN1106_s1383B000093	2
Serpin	BN1106_s3864B000104	2
Tropinone reductase;3-oxoacyl-[acyl-carrier-protein] reductase; 2-deoxy-d-gluconate 3-dehydrogenase	BN1106_s1612B000139	2
Ubiquitin-protein ligase BRE1	BN1106_s208B000185	4
UTP-glucose-1-phosphate uridylyltransferase	BN1106_s244B000349	3
Zinc binding dehydrogenase;Trans-2-enoyl-CoA reductase	BN1106_s4810B000086	2
*Biological Process: Cellular component organization/biogenesis (GO:0071840)*		
Calponin	BN1106_s773B000382	2
*Biological Process: Cellular process (GO:0009987)*		
Dynein light chain	BN1106_s1444B000096	2
*No Gene Ontology characterisation*		
Uncharacterised	BN1106_s263B000603	2
Venom allergen-like (VAL) 11 protein	BN1106_s1956B000118	2

The abundance of mannosylated glycoproteins revealed by our lectin array and lectin blot analysis was supported by the high number of proteins identified by proteomic analysis of Man-FhTeg. A total of 341 proteins were identified based on at least two unique peptides using ConA-, LCA-, or GNL-lectin affinity purified fractions (209, 185, or 301, respectively). As expected, the three Man-binding lectins isolated similar subsets of glycoproteins (222 shared hits; 65%), though a number of glycoproteins were unique to each subset (Fig. 5*B*). Although each lectin did not show any enrichment for a particular type of protein, several important *Fasciola* proteins were found uniquely in one or other fraction. For example, cathepsin L and cathepsin B cysteine proteases were identified in the ConA fraction, an asparaginyl endopeptidase (legumain) in the LCA fraction and a zeta class gluthatione S-transferase (GST) in the GNL fraction (supplemental Table S5).

To define the most abundant glycoproteins in the parasite tegument, the 50 most represented proteins in each of the ConA-, LCA-, or GNL-lectin affinity purified fractions was compiled, based on the number of unique peptides, and resulted in a total of 74 glycoproteins; these were then categorised according to their GO classifications (Table IV, supplemental Tables S5–S7. Twenty eight glycoproteins were identified as being particularly abundant in all three mannose-rich glycoprotein preparations, which included structural proteins, metabolic enzymes, heat shock proteins and cysteine proteases. Of the remaining 46 glycoproteins, 31 glycoproteins were only identified in one of the three preparations, indicating that these proteins exhibit lectin-specific glycoforms. Although not particularly abundant, a number of proteins were identified with GO classifications associated with glycosylation pathways (GO:0006013: mannose metabolic process; GO:0006491: *N*-linked oligosaccharide processing) and the immune response (GO:0071353: Cellular response to IL-4; GO:0030593; Neutrophil chemotaxis; GO:0007159: Leukocyte cell-cell adhesion).

**Table IV:** Representation of the top proteins identified by mannose-specific lectin affinity chromatography, annotated using the F. hepatica genome, grouped by Gene Ontology classification

Description	Identifier	Unique Peptide		
		ConA	LCA	GNL
*Molecular Function: Binding (GO:0005488)*				
Actin	BN1106_s2907B000133	14	8	12
Alpha actinin	BN1106_s4069B000247	24	7	24
Basement membrane-specific heparan sulfate proteoglycan core protein	BN1106_s25B000189	11	50	33
Calnexin	BN1106_s553B000158	2	6	8
Calreticulin	BN1106_s2673B000071	9	3	6
Filamin	BN1106_s1515B000336	9	0	13
Filamin	BN1106_s296B000186	9	0	3
Fimbrin/Plastin	BN1106_s1403B000129	13	9	15
Gelsolin/Severin	BN1106_s2349B000188	11	8	12
HSP70	BN1106_s309B000234	13	10	14
HSP70/HSP105	BN1106_s2091B000373	9	3	16
HSP90	BN1106_s1320B000236	7	7	12
Myosin	BN1106_s323B000258	9	11	14
Myosin	BN1106_s3182B000117	0	8	15
Tegumental calcium binding EF hand protein	BN1106_s214B000744	7	3	8
Telomerase protein component 1	BN1106_s306B000267	10	10	10
200kDa GPI anchored surface glycoprotein	BN1106_s168B000275	5	15	16
*Molecular Function: Catalytic activity (GO:0003824)*				
Acetate:succinate CoA transferase	BN1106_s11911B000016	7	4	9
Aldehyde dehydrogenase	BN1106_s645B000322	7	0	6
Alpha mannosidase	BN1106_s666B000200	0	10	3
Calpain	BN1106_s204B000249	12	8	17
cAMP dependent protein kinase	BN1106_s2316B000078	4	0	10
Cathepsin A (Carboxypeptidase C)	BN1106_s1241B000264	5	8	7
Cathepsin B10	BN1106_s1840B000150	4	6	5
Cathepsin L1	BN1106_s8490B000026	7	7	7
Cathepsin L2	BN1106_s8098B000020	12	11	12
Cathepsin L5	BN1106_s4636B000039	8	8	7
Enolase	BN1106_s3227B000227	8	5	12
ER calcistorin/Protein disulphide isomerase	BN1106_s2763B000063	19	10	5
Fructose bisphosphate aldolase	BN1106_s4469B000065	12	10	12
Galactosidase alpha	BN1106_s1241B000260	8	10	12
Galactosidase beta	BN1106_s5248B000014	6	7	2
Glutamate dehydrogenase	BN1106_s5767B000030	10	11	12
Glutathione dehydrogenase	BN1106_s50B000678	8	3	8
GST sigma class	BN1106_s1081B000242	6	5	8
Hexokinase A	BN1106_s175B000200	5	4	9
Lysosomal Pro X carboxypeptidase	BN1106_s1620B000120	0	7	5
Lysosomal Pro X carboxypeptidase	BN1106_s3518B000132	4	8	11
Malic enzyme	BN1106_s233B000262	12	8	16
Mut protein	BN1106_s3452B000178	7	5	13
Nardilysin	BN1106_s2211B000138	7	9	23
Paramyosin	BN1106_s1922B000122	11	8	9
Propionyl CoA carboxylase	BN1106_s1551B000468	12	4	7
Propionyl CoA carboxylase	BN1106_s1252B000359	16	12	19
Phosphatidylcholine-sterol acyltransferase	BN1106_s4998B000033	3	9	6
Phosphoenolpyruvate carboxykinase	BN1106_s246B000252	13	12	19
Protein disulphide isomerase	BN1106_s4999B000041	7	7	3
Pyrroline 5 carboxylate reductase	BN1106_s1098B000219	8	2	4
Pyruvate carboxylase	BN1106_s1091B000125	7	4	14
Serpin	BN1106_s3864B000104	5	6	5
Serpin	BN1106_s1727B000096	5	6	8
Sulfhydryl oxidase	BN1106_s207B000270	4	8	7
Succinate dehydrogenase	BN1106_s1501B000239	7	4	7
2 oxoglutarate dehydrogenase	BN1106_s425B000529	15	7	27
14–3-3 protein	BN1106_s3904B000042	5	3	9
14–3-3 protein	BN1106_s686B000273	10	8	8
*Molecular Function: Molecular function regulator (GO:0098772)*				
Annexin	BN1106_s945B000218	7	8	7
Annexin	BN1106_s819B000364	10	8	10
Multi-domain cystatin (cys1)	BN1106_s1612B000138	16	44	44
*Molecular Function: Structural molecule activity (GO:0005198)*				
Collagen type XV alpha	BN1106_s26B000447	14	18	15
Spectrin	BN1106_s2351B000181	5	12	28
Spectrin	BN1106_s4255B000066	8	18	50
Tubulin beta-3b	BN1106_s4860B000047	9	8	9
Tubulin alpha-2	BN1106_s925B000543	5	4	9
*Biological Process: Biological adhesion (GO:0007155)*				
Laminin	BN1106_s656B000154	0	7	0
*Biological Process: Localization (GO:0051179)*				
Restin/Dynactin 1	BN1106_s980B000151	0	6	8
*No Gene Ontology characterisation*				
DM9 domain containing protein	BN1106_s5689B000026	11	10	9
Tropomyosin	BN1106_s4130B000080	5	8	7
Vacuolar protein sorting 26 (vps26)	BN1106_s2566B000129	3	5	10
Uncharacterised	BN1106_s8038B000016	7	7	8
Uncharacterised	BN1106_s1647B000242	8	14	4
Uncharacterised	BN1106_s6006B000040	9	5	5
Uncharacterised	BN1106_s6821B000024	4	9	5

**Fig. 6.**
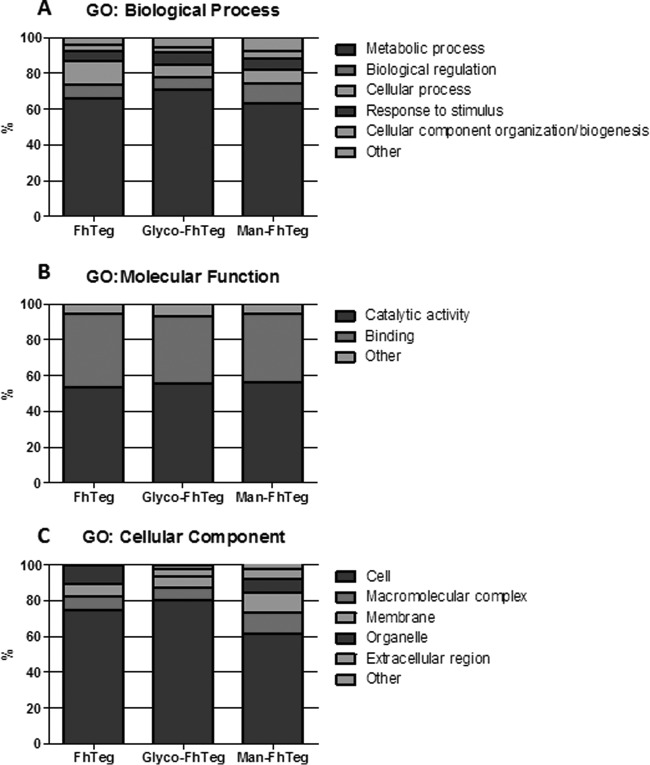
**Comparative Gene Ontology analysis of the protein components of FhTeg, Glyco-FhTeg and Man-FhTeg preparations.** Stacked column charts show the percentage distribution of proteins identified in FhTEg, GlycoFhTeg and Man-FhTeg fractions classified by Biological Process, Molecular Function and Cellular Compartment.

All 341 proteins identified in Man-FhTeg (supplemental Table S5–S7) were analyzed for the presence of potential glycosylation motifs. This analysis revealed that 34 proteins had no putative glycosylation sites (51 when higher stringency (0.75) was applied to the N-glycosylation predictive threshold). Cathepsins L2 and L5, fatty acid binding protein Fh2 and Fh3 are included among the proteins with no putative glycosylation sites. This data suggests that these nonglycosylated proteins could be coeluted with mannosylated proteins as part of multimolecular complexes. Interestingly, several nonglycosylated proteins identified in the Man-FhTeg preparation belonged to the proteosome complex (GO:0019773). Glycoproteins in both the Man- and Glyco-FhTeg fractions exhibit a wide variety of subcellular locations observed, which is not surprising because the surface tegument is composed not only of membrane-associated proteins but also cytoplasmic, cytoskeletal and microtubule-associated proteins (10) (Fig. 6).

## DISCUSSION

This study examining the glycosylation of *F. hepatica* surface tegument is the most extensive lectin-based investigation to date. Here, we found an abundance of carbohydrate motifs on the outer aspects of the parasite surface, whereas lectin fluorescence microscopy experiments, conducted under nonpermeabilizing conditions, enabled the remarkable observation of intricate details of the tegumental morphology, especially spines and spinelets that are not often revealed in standard fluorescence micrographs. Plant lectins have been previously used to probe *F. hepatica* preparations and revealed a complex collection of exposed oligosaccharide structures in the tegument (27–29) but the molecular nature of these presentations remains enigmatic.

FhTeg was comprised of a rich mixture of glycoconjugates. However, we discovered that most proteins are predominantly modified by oligomannose glycans that are widely distributed on the spines, suckers and tegumental coat of *F. hepatica* and lectin blot analysis supported the abundance of these N-glycosylated proteins. It is likely that glycoproteins on the tegument are modified with a mixture of *N*-linked oligosaccharide structures as different lectins tested in the analysis bound to protein bands of similar molecular size. Oligosaccharide structures with terminal GlcNAc and GalNAc were distributed widely on the anatomical features of the parasite surface, although a smaller panel of glycoproteins carrying these residues were identified by lectin blot. In contrast, Fuc, Gal, and α(2–3)-linked sialic acid residues were distributed in discrete anatomical regions only. The sialic acid-binding proteins MAL II and SNA-I recognized ligands predominantly located on the parasite surface, corroborating the previous observation of sialylated glycoproteins in the *F. hepatica* tegument (12, 29). This is of special interest as helminths are believed to lack the biosynthetic machinery required for sialylation and raises the possibility that FhTeg sialic acid-containing oligosaccharides are derived from host glycoproteins as a form of immune defense, as also suggested for blood-sucking adult *Ixodes ricinus* ticks although we have no evidence to support this (30).

Tegumental proteins of *F. hepatica* represent a very complex biological matrix, but the extent of its complexity has only recently become clearer because of the emergence of proteomic and glycomic methods, together with the availability of extensive transcriptomic and genomic data (29). In this study, proteomic analysis of the FhTeg, Glyco-FhTeg and Man-FhTeg protein fractions identified a total of 369 proteins. A small subset of host proteins were also identified, mainly those associated with the host blood and immune system. The identification of host immunoglobulins is consistent with proteomic analysis of *Schistosoma mansoni* tegument (31). Braschi and colleagues inferred that although previous studies have shown that schistosomes are able to envelop themselves in host proteins, the host proteins are present in small concentration that are enriched during the streptavidin affinity purification protocol. The enrichment of FhTeg by lectin affinity chromatography also aided in the identification a greater number of *F. hepatica* specific-proteins across three preparations than the analysis of a single set alone. And, despite these differences, they shared a similar gene ontology profile. Comparing the output of our proteomic study with other reports, nearly 60% of the proteins were identified previously using *F. hepatica* preparations obtained by Nonidet P-40 extraction or freeze-thaw-vortex (4, 5, 6). In particular, when compared with the most recent and comprehensive proteomic study by Hacariz *et al.* (2012) that identified 444 proteins, their analysis revealed more putative proteins isoforms with 75 additional proteins (32). Similarly, comparative analysis based on putative protein annotation, with schistosome tegument proteomic datasets (33, 34), revealed that ∼50% of the proteins overlapped with our study, including several proteins associated with structural and motor function, binding, kinase activity and enzymatic activity. Differences between these various studies likely reflects the variability between parasite isolates, methods for protein isolation and the different databases used for analysis of MS data. In the present study, gene models were reliably identified by our recently sequenced *F. hepatica* draft genome (4, 5, 6, 35).

The surface tegument consists of a cellular syncytium covered with a glycocalyx that is continually replaced by highly active subtegumental cells that send vesicles to the surface to maintain its integrity and defend against host immune attack (12). Thus it can be viewed as a dynamic tissue and, hence, it is not surprising that several proteins of mitochondrial origin were identified in FhTeg and enriched in the Glyco-FhTeg and Man-FhTeg fractions, which is consistent with previous studies on tegumental and surface protein preparations from *F. hepatica* (5, 6). The *in silico* prediction of N-glycosylation sites revealed the presence of putative *N*-linked glycosylation for all observed mitochondrial proteins except phosphate carrier protein, voltage-dependent anion-selective channel protein 2 and ATP:ADP antiporter. Although glycosylation of mitochondrial proteins is generally considered rare, recent studies have shown the presence of *N*-linked glycosylation in mitochondria and on proteins with mitochondrial functions in rats and cattle (36, 37).

The success of helminth parasites such as *F. hepatica* has been attributed to their ability to subvert the immune response of their host (38) and they do this by excreting and secreting a battery of molecules (often-termed ES products). Proteomic analysis of the ES products (FhES) of adult *F. hepatica* suggested that some molecules are released using classical secretory signal pathways mainly from the parasite digestive system and by nonclassical means in the form of blebbing or the release of vesicles from the tegument (39). More recently, we have characterized the immune modulatory properties of *F. hepatica* tegument demonstrating similarities to the FhES potent Th1 immune suppressive properties (13). In contrast, although FhES induces adaptive Th2/Treg cell responses, FhTeg induces anergenic-like immune responses that can be reversed by the addition of IL-2 to culture (40).

*F. hepatica* excretory-secretory products and antigens released from other helminths (41–43) were shown to interact with C-type-lectin receptors (CLR) pointing to the contribution of glycoconjugates to their immune modulatory properties. Although FhTeg glycoproteins engage with the mannose receptor (MR) its immunomodulatory activity is retained even in the absence of MR, suggesting the involvement of other CLRs (44). This result was surprising given that FhTeg appears to include a large group of highly mannosylated proteins. Although FhTeg activated DCs induce anergenic T-cells and MR is critical in this process we have yet to determine if FhTeg interaction is essential to the phenotypic development of FhTeg activated DCs that express high levels of MR (34). The involvement of other CLRs, such as DC-SIGN, MGL, and Dectin-2 is most extensively studied for *S. mansoni* soluble egg antigens (45, 46) demonstrating that glycoconjugates interact with a number of CLR's on innate immune cells with a synergistic effect (46). Although currently, little is known about CLR-targeting by FhTeg it is possible given that there is a wide variety of carbohydrate motifs that are similar to other helminths and are involved in the fluke's immune regulatory properties through the potential host recognition of parasite glycoproteins (44).

The immune modulatory molecules of FhTeg have yet to be identified, however, this analysis revealed a number of glycoproteins that may be potential candidates some of which have not been previously reported. One such molecule, which was found in *F. hepatica* exosomes (39), is the receptor for tetraspanin CD63, a surface-associated membrane protein involved in innate immune cell activation, adhesion and differentiation (47–49). A second molecule, TRIL (TLR4 interactor with leucine-rich repeats), is a recently discovered accessory protein of TRL4 and TLR3 signaling complexes (50, 51) that is highly expressed in tissues from different species (49). TRIL can bind directly to LPS (51), which could explain how FhTeg prevents LPS-driven responses in dendritic cells and mast cells (13, 14). Metalloendopeptidase nardilysin, a peptidase that was shown to be ubiquitously localized in several mammalian organ systems (52) is involved in TNFα shedding from immune cells through the binding and activation of TNFα-converting enzyme (53). It is also a putative shedding enzyme for VTCN-1 (V-set domain-containing T cell activation inhibitor-1), an inhibitory molecule of T-cell activation that has an important role in the balance between abnormal T-cell activation and T-cell anergy. *F. hepatica* nardilysin-like molecules may act as a decoy preventing the binding and activation of TNFα-converting enzyme or may contribute to T-cell imbalance leading to T-cell anergy, which is associated with FhTeg (40).

Although FhTeg glycoproteins are highly decorated with oligosaccharides, we do not know if these oligosaccharides are important to the immune response during infection. Anti-oligosaccharide antibodies are present in sera from Schistosoma infected humans, monkeys and mice (54, 55) and these antibodies identified a number of antigenic oligosaccharides including LDNF LDN and Le^X^ that are found on the surface tegument of adult *S. mansoni* and schistosomula (56, 57). Antigenic oligosaccharides were also reported in infection with metacestodes, cestodes, and filarial nematodes (56). These oligosaccharides can be unique to helminth species or structures can also be shared with plants and invertebrates making them viable vaccine candidates. Identification of the most potent immunogenic glycoconjugates could lead to the development of novel carbohydrate based vaccine candidates. *F. hepatica* mannosylated glycoproteins induced significant protection against *S. mansoni* infected mice however it is yet to be determined if the glycoconjugates are important to the development of immunity (58). Similarly, Venom Allergen-like II protein (59, 60) and enolase (61, 62), which were also identified in worm tegument, have also been posited as potential vaccine candidates. Consistent with preparations of FhES, several putative vaccine candidates were identified in our three tegumental preparations. Interestingly, cathepsin B and paramyosin were only identified in the Man-FhTeg preparation, which suggests that these proteins are glycosylated with mannose oligosaccharides.

Despite the advancement of our understanding of helminth pathogens, no commercial vaccine is available to prevent helminth infection. “Barbervax” is a multicomponent complex of natively purified gut-derived proteins/glycoproteins ofthe intestinal nematode *Hemonchus contortus* (barbervax.com.au) that was recently released as a commercial vaccine. However, it has not been determined if the carbohydrate residues as important for immunity. In contrast there are a number of commercially successful carbohydrate based bacterial vaccines that are derived from natural and synthetic sources that utilize nonspecific conjugation methods to carrier proteins (63). Carbohydrate-based vaccines for helminth therefore could be a reality. However, the factors that inhibit the development of the vaccine are the complexity of helminth biology and its immune modulatory properties. Given that *F. hepatica* and other helminth proteins are extensively coated with glycoconjugates, the failure of helminth-derived vaccines to date may be because of the lack of effective antibody responses against these molecules that play such an important role in helminth biology and immune modulatory properties. This study reveals the complex matrix of *F. hepatica* tegumental proteins by providing novel insights regarding their putative glycosylation and the importance of this work is therefore raises questions to the role these glycoconjugates in host-parasite interactions and in glycoprotein based helminth vaccine.

### 

#### 

##### Data access statement

The mass spectrometry proteomics data have been deposited to the ProteomeXchange Consortium via the PRIDE (64) partner repository with the dataset identifier PXD003911 and 10.6019/PXD003911.

## Supplementary Material

Supplemental Data

## Footnotes

^1^ The abbreviations used are:

CTRs: C-type lectin receptors
DCs: Dendritic Cells
FhTeg: *Fasciola hepatica* Tegumental Antigens
MAPK: Mitogen-activated protein kinases
NK-kB: Nuclear factor kappa B
SOCS3: Suppressor of cytokine signalling (SOCS) 3
STAT: signal transducers and activators of transcription3
TLR: Toll-like receptor
TRIL: TLR4 interactor with leucine-rich repeats
VTCN-1: V-set domain-containing T cell activation inhibitor-1
WHO: World Health Organisation.
