# Supplementary material for: Fasciola hepatica Surface Tegument: Glycoproteins at the Interface of Parasite and Host
Source: Mol Cell Proteomics. 2016 Jul 27;15(10):3139–53. doi: 10.1074/mcp.M116.059774 (PMC5054340; doi:10.1074/mcp.M116.059774)
Supplement: Supplemental Data [file supp_15_10_3139__index.html]

Fasciola hepatica surface tegument: glycoproteins at the interface of parasite and host — Fasciola hepatica Surface Tegument: Glycoproteins at the Interface of Parasite and Host — Fasciola hepatica Tegumental Oligosaccharides — Supplemental Data 

# *Fasciola hepatica* Surface Tegument: Glycoproteins at the Interface of Parasite and Host

## Supplemental Data

- SM\_Figure (.pptx, 2.5 MB) - SM\_Figure
- Table S1 (.docx, 31 KB) - Table S1
- Supplemental Table 2 (.xlsx, 42 KB) - Supplemental Table 2
- Supplemental Table 3 (.xlsx, 85 KB) - Supplemental Table 3
- Supplemental Table 4 (.xlsx, 16 KB) - Supplemental Table 4
- Supplemental Table 5 (.xlsx, 15 KB) - Supplemental Table 5
- Supplemental Table 6 (.xlsx, 19 KB) - Supplemental table 6
- Supplemental table 7 (.xlsx, 27 KB) - Supplemental table 7
