# Supplementary material for: Fasciola hepatica Surface Tegument: Glycoproteins at the Interface of Parasite and Host
Source: Mol Cell Proteomics. 2016 Jul 27;15(10):3139–53. doi: 10.1074/mcp.M116.059774 (PMC5054340; doi:10.1074/mcp.M116.059774)
Supplement: Supplemental Data [file 10.1074_M116.059774_mcp.M116.059774-1.pptx]

## Slide 1
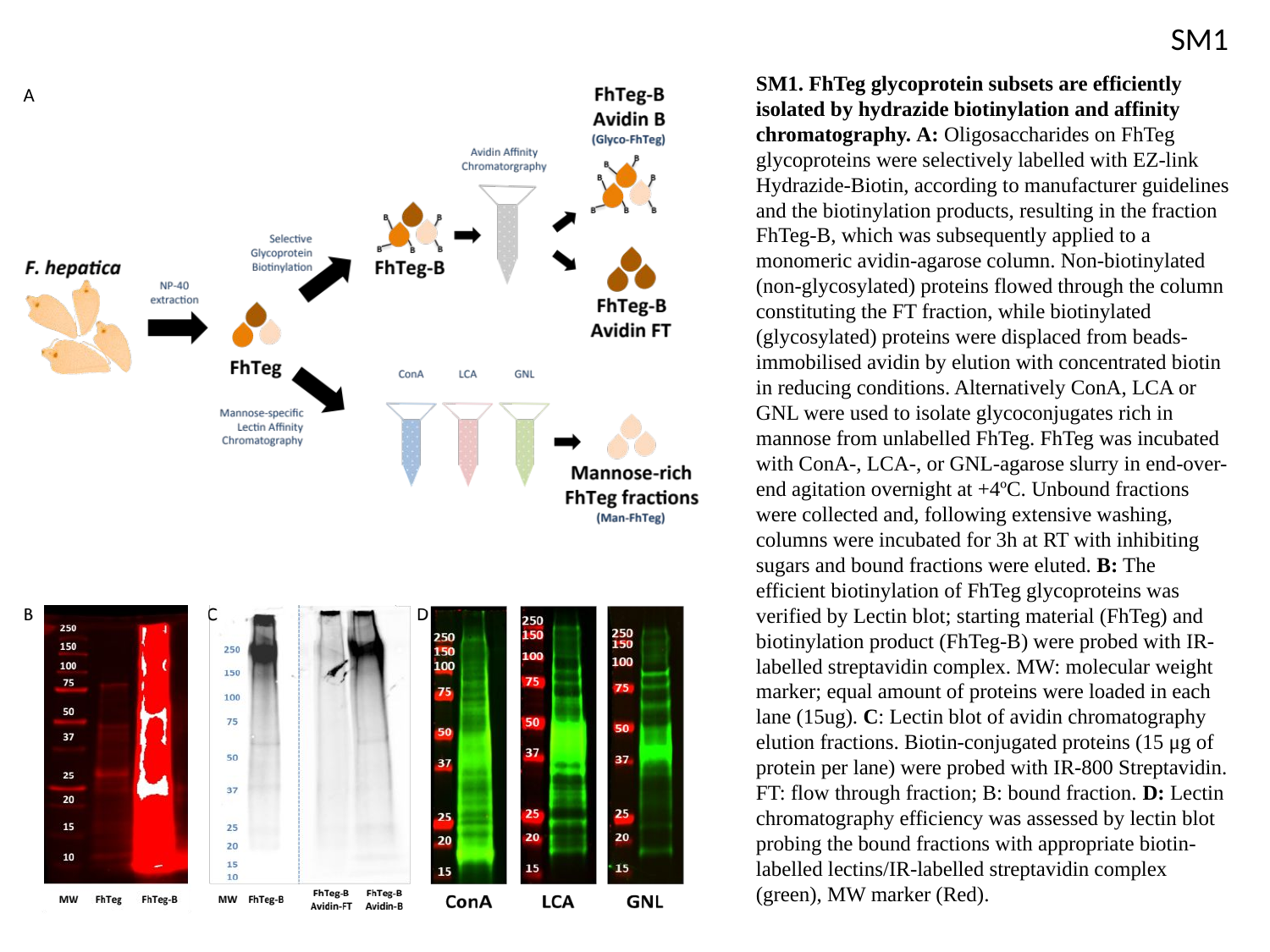

SM1
SM1. FhTeg glycoprotein subsets are efficiently isolated by hydrazide biotinylation and affinity chromatography. A: Oligosaccharides on FhTeg glycoproteins were selectively labelled with EZ-link Hydrazide-Biotin, according to manufacturer guidelines and the biotinylation products, resulting in the fraction FhTeg-B, which was subsequently applied to a monomeric avidin-agarose column. Non-biotinylated (non-glycosylated) proteins flowed through the column constituting the FT fraction, while biotinylated (glycosylated) proteins were displaced from beads-immobilised avidin by elution with concentrated biotin in reducing conditions. Alternatively ConA, LCA or GNL were used to isolate glycoconjugates rich in mannose from unlabelled FhTeg. FhTeg was incubated with ConA-, LCA-, or GNL-agarose slurry in end-over-end agitation overnight at +4ºC. Unbound fractions were collected and, following extensive washing, columns were incubated for 3h at RT with inhibiting sugars and bound fractions were eluted. B: The efficient biotinylation of FhTeg glycoproteins was verified by Lectin blot; starting material (FhTeg) and biotinylation product (FhTeg-B) were probed with IR-labelled streptavidin complex. MW: molecular weight marker; equal amount of proteins were loaded in each lane (15ug). C: Lectin blot of avidin chromatography elution fractions. Biotin-conjugated proteins (15 μg of protein per lane) were probed with IR-800 Streptavidin. FT: flow through fraction; B: bound fraction. D: Lectin chromatography efficiency was assessed by lectin blot probing the bound fractions with appropriate biotin-labelled lectins/IR-labelled streptavidin complex (green), MW marker (Red).
A
