# Supplementary material for: Fasciola hepatica Surface Tegument: Glycoproteins at the Interface of Parasite and Host
Source: Mol Cell Proteomics. 2016 Jul 27;15(10):3139–53. doi: 10.1074/mcp.M116.059774 (PMC5054340; doi:10.1074/mcp.M116.059774)
Supplement: Supplemental Data [file 10.1074_M116.059774_mcp.M116.059774-2.docx]

**Table S1.** Lectins printed, their abbreviations, binding specificities, simple print sugars (1 mM) and the supplying company for the lectin microarray.
